# Expression of Tumor Necrosis Factor-Alpha-Mediated Genes Predicts Recurrence-Free Survival in Lung Cancer

**DOI:** 10.1371/journal.pone.0115945

**Published:** 2014-12-30

**Authors:** Baohua Wang, Ning Song, Tong Yu, Lianya Zhou, Helin Zhang, Lin Duan, Wenshu He, Yihua Zhu, Yunfei Bai, Miao Zhu

**Affiliations:** 1 Department of Thoracic Surgery, The Second Hospital of HeBei Medical University, Shijiazhuang, 050000, China; 2 Department of Respiratory Medicine, The Second Hospital of HeBei Medical University, Shijiazhuang, 050000, China; 3 Nanjing Decode Genomics Biotechnology Co., Ltd., Nanjing, 210019, China; 4 School of Biological Sciences and Medical Engineering, Southeast University, Nanjing, 210096, China; 5 College of Information Science and Technology, Nanjing Agricultural University, Nanjing, 210095, China; Istituto dei tumori Fondazione Pascale, Italy

## Abstract

In this study, we conducted a meta-analysis on high-throughput gene expression data to identify TNF*-*α-mediated genes implicated in lung cancer. We first investigated the gene expression profiles of two independent TNF-α/TNFR KO murine models. The EGF receptor signaling pathway was the top pathway associated with genes mediated by TNF-α. After matching the TNF-α-mediated mouse genes to their human orthologs, we compared the expression patterns of the TNF-α-mediated genes in normal and tumor lung tissues obtained from humans. Based on the TNF-α-mediated genes that were dysregulated in lung tumors, we developed a prognostic gene signature that effectively predicted recurrence-free survival in lung cancer in two validation cohorts. Resampling tests suggested that the prognostic power of the gene signature was not by chance, and multivariate analysis suggested that this gene signature was independent of the traditional clinical factors and enhanced the identification of lung cancer patients at greater risk for recurrence.

## Introduction

Tumor necrosis factor-alpha (TNF-α) is a pleiotropic inflammatory cytokine involved in systemic inflammation that stimulates the acute phase reaction. This cytokine affects most human organs and is involved in a variety of biological processes, including cell proliferation, differentiation, apoptosis, lipid metabolism, and coagulation [1], [2], [3]. TNF-α also serves as a mediator in various pathologies, such as septic shock, transplantation rejection, multiple sclerosis, diabetes, rheumatoid arthritis, trauma, malaria, meningitis, and adult respiratory distress syndrome [4].

The role of TNF-α in human cancers is more complicated [5], [6]. TNF-α has a dual role in tumor induction and progression [7]. TNF-α can facilitate the generation and maintenance of antitumor immune responses through the activation of natural killer cells and CD8 T cells [7], [8]. TNF-α also inhibits tumor-induced vascularization by damaging the tumor-associated vasculature [9]. Furthermore, TNF-α can directly affect tumor cells by increasing lysosomal enzymes and inducing cytochrome c release from the mitochondria and apoptosis [10].

Although TNF-α has antitumor activity, there is growing evidence that suggests that endogenous TNF-α acts as a tumor promoter. TNF-α has been known to contribute to chronic inflammation and promote tumor formation, growth and metastasis [7], [11]. It has also been observed that TNF-α knockout (KO) mice were more resistant to chemical carcinogenesis of skin tumors [12], [13]. Additionally, mice deficient in TNF receptor type 1 (TNFR-1) and TNF receptor type 2 (TNFR-2) were resistant to the development of skin tumors [14], and knockdown of TNF-α in ovarian cancer cell lines led to diminished growth and vascular density [15]. More recently, Pitroda et al. examined the role of TNF-α-mediated stromal inflammation in tumor growth. They found that disruption of stromal TNF-α signaling significantly impaired the growth of tumors in TNF-α receptor (TNFR) KO mice compared to that in wild-type (WT) mice [16]. Based on this experimental model of TNF-α-mediated inflammation and high-throughput gene expression data, they also developed a prognostic gene signature that predicted overall survival in human cancers [16].

Lung cancer is the most frequently diagnosed cancer and is the leading cause of cancer death in males, comprising 17% of the total new cancer cases and 23% of the total cancer deaths [17]. In this study, we conducted meta-analysis on high-throughput expression microarray data to identify the TNF*-*α-associated genes that were implicated in lung cancer. First, we identified the genes potentially regulated by TNF-α and TNFRs. Using two independent microarray datasets, we characterized the common genes dysregulated in TNF-α KO and TNFR KO mice. We regarded these genes as TNF-α-associated genes. Second, we developed a prognostic gene signature derived from the TNF-α-associated genes, and we matched the TNF-α-associated genes in mouse to their human orthologs. We then compared the expression of human TNF-α-associated genes in normal and tumor tissues of two lung cancer cohorts. Seventeen TNF-α-associated genes were identified as being commonly differentially expressed between the two groups; therefore, these genes composed a multi-molecular cancer outcome predictor. This molecular signature effectively predicted recurrence-free survival in lung cancer and was independent of the standard clinical and pathological prognostic factors.

## Results and Discussion

### TNF-α-associated genes

We first investigated the genes potentially regulated by TNF-α and its receptors. TNF-α binds two receptors, TNFR1 and TNFR2. TNFR1 is expressed in most tissues, whereas TNFR2 is found only in cells of the immune system. Two independent microarray datasets containing gene expression information for both WT and TNF-α/TNFR KO mice were collected from the Gene Expression Omnibus (GEO) database [18]. We compared the gene expression patterns of gastric tumors of WT and TNF-α KO mice (GEO accession ID: GSE43145) and of melanomas of WT and TNFR KO mice (GEO accession ID: GSE33253) [16]. At a <5% false discovery rate (FDR), 390 genes were commonly up-regulated compared with WT mice in both datasets (S1 Table). In contrast, 305 genes were found to be commonly down-regulated compared with WT mice (S1 Table). We deemed these dysregulated genes TNF-α-mediated genes. Pathway analysis using the PANTHER database [19] indicated that the top pathway associated with these dysregulated genes was the “EGF receptor signaling pathway” (*P* = 0.029 by Fisher’s exact test) (S2 Table), which suggests a strong relationship between TNF-α and epidermal growth factor receptor (EGFR). EGFR is a member of the ErbB family of receptors. Alteration in EGFR expression may result in cancer [20], including lung cancer, anal cancers, and glioblastoma multiforme. TNF-α is a central regulator of multiple inflammatory signaling pathways, and one important target of TNF-α may be the signaling pathway downstream of EGFR [21]. In fact, TNF-α has been shown to induce EGFR transactivation in a variety of cells [22], [23], [24].

**Figure 1:**
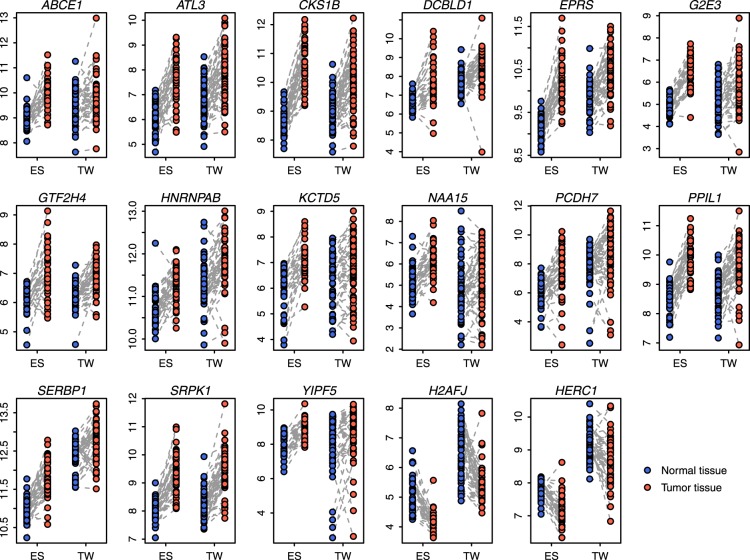
Comparison of the expression of the 17-gene signature in normal and tumor tissues. Paired normal and tumor tissues from lung cancer patients in the ES and TW cohorts were included in the comparison. Y-axis: log2-transformed expression values.

To determine whether TNF-α-mediated genes derived from mice were relevant to human cancers, we matched the TNF-α-mediated mouse genes to 651 distinct human orthologs. Next, we analyzed the expression patterns of these TNF-α-mediated human genes in lung cancer. We explored the difference in gene expression between normal and tumor tissues in two independent lung cancer cohorts from Spain (ES [GEO accession ID: GSE18842]) [25] and Taiwan (TW [GEO accession ID: GSE19804]) [26]. A paired t-test was used to identify the differentially expressed genes between the normal and tumor tissues (Fig. 1). In total, 232 TNF-α-mediated genes were identified as being commonly differentially expressed between the normal and tumor tissues (adjusted *P*<0.05 and fold change >1.25) in both cohorts (S3 Table). One hundred and twenty-nine TNF-α-mediated genes were up-regulated in tumor tissues, while 103 TNF-α-mediated genes were down-regulated (S3 Table). To investigate in which functional categories these genes fall, we conducted gene ontology analysis using the GO database [27]. We found that these genes were significantly enriched by GO biological process terms, such as “macromolecule catabolic process”, “tRNA aminoacylation”, and “ubiquitin-dependent protein catabolic process” (S4 Table).

### A 17-gene signature predicts recurrence-free survival in lung cancer

We hypothesized that the 232 differentially expressed TNF-α-mediated genes might predict tumor outcome in lung cancer. Univariate Cox proportional hazards regression against recurrence-free survival was conducted across the 232-gene set in a training cohort from Korea (KR [GEO accession ID: GSE8894]) [28]. Only the genes with absolute values of the Wald statistic (ratio of the regression coefficient to its standard error) larger than two were retained. In total, we identified 17 TNF-α-mediated genes associated with lung cancer recurrence-free survival in the training cohort. We designated the 17 TNF-α-mediated genes as the 17-gene signature (Fig. 1 and Table 1).

**Table 1:** 17-gene signature.

Gene symbol	Chromosome	Gene description	Weight
*ABCE1*	4	ATP-binding cassette, sub-family E (OABP), member 1	3.24
*ATL3*	11	Atlastin GTPase 3	2.21
*CKS1B*	1	CDC28 protein kinase regulatory subunit 1B	2.10
*DCBLD1*	6	Discoidin, CUB and LCCL domain-containing 1	3.03
*EPRS*	1	Glutamyl-prolyl-tRNA synthetase	2.34
*G2E3*	14	G2/M-phase specific E3 ubiquitin protein ligase	2.35
*GTF2H4*	6	General transcription factor IIH, polypeptide 4, 52 kDa	2.35
*HNRNPAB*	5	Heterogeneous nuclear ribonucleoprotein A/B	3.42
*KCTD5*	16	Potassium channel tetramerization domain-containing 5	2.53
*NAA15*	4	N(alpha)-acetyltransferase 15, NatA auxiliary subunit	3.07
*PCDH7*	4	Protocadherin 7	2.33
*PPIL1*	6	Peptidylprolyl isomerase (cyclophilin)-like 1	2.03
*SERBP1*	1	SERPINE1 mRNA-binding protein 1	2.85
*SRPK1*	6	SRSF protein kinase 1	2.42
*YIPF5*	5	Yip1 domain family, member 5	2.04
*H2AFJ*	12	H2A histone family, member J	−2.13
*HERC1*	15	HECT and RLD domain-containing E3 ubiquitin proteinligase family member 1	−2.31

**Figure 2:**
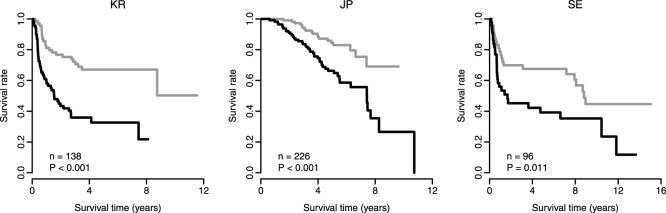
Kaplan-Meier curves of recurrence-free survival. The recurrence score of the 17-gene signature predicts poor clinical outcome in lung cancer. The black curves are for the 17-gene-signature-positive patients, while the gray curves are for the 17-gene-signature-negative patients. The 17-gene-signature-positive patients were defined as those having a recurrence score greater than the group median score. *P*-values were calculated using log-rank tests for the differences in survival. The left panel shows the training cohort (KR), while the middle and right panels show the validation cohorts (JP and SE).

We applied a scoring system to assign each patient a recurrence score that was a linear combination of the expression of the 17-gene signature weighted by the coefficients obtained from the training cohort (see the Methods for details) [16], [29], [30], [31]. The weight for each gene is listed in Table 1. The 17-gene positive patients were defined as those having a recurrence score greater than the group median. As expected, there was a significantly reduced recurrence-free survival for the 17-gene-positive patients in the training cohort (Fig. 2). The 17-gene positive patients had a significantly increased risk for recurrence of 2.95-fold in the KR cohort (Table 2).

**Table 2:** Univariate Cox proportional hazards regression of survival by the 17-gene status.

Cohort	Type	Sample size	Hazard ratio	95% Confidence interval	*P*-value
KR	Training	138	2.95	(1.77, 4.92)	3.2×10^−5^
JP	Validation	226	2.67	(1.56, 4.58)	3.5×10^−4^
SE	Validation	96	2.08	(1.17, 3.69)	1.2×10^−2^

We next investigated the prognostic power of the recurrence score that was determined using the 17-gene signature in independent validation cohorts. Two validation cohorts from Japan (JP [GEO accession ID: GSE31210]) [32] and Sweden (SE [GEO accession ID: GSE37745]) [33] were collected. Kaplan-Meier survival analysis demonstrated a significantly reduced recurrence-free survival for the 17-gene-positive patients in the validation cohorts (*P* = 5.4×10^−5^ for the JP cohort and *P* = 0.011 for the SE cohort by log-rank test) (Fig. 2). Univariate Cox proportional hazards regression indicated that 17-gene-positive patients had a significantly increased risk for recurrence of 2.67-fold in the JP cohort and of 2.08-fold in the SE cohort (Table 2). These findings indicated that the 17-gene signature is predictive of recurrence-free survival in lung cancers.

We also investigated the prognostic power for each gene within the 17-gene signature in the JP cohort, which is the largest cohort in this study. The patients were stratified into two groups according to the expression level of each gene, using the median as a cutoff. We identified three genes, *HNRNPAB*, *PPIL1*, and *SRPK1*, which can be used to predict recurrence-free survival individually (adjusted *P*<0.05 by log-rank test) (S1 Fig.).

Actually, among the 17-gene set, *ABCE1*, *CKS1B*, *HNRNPAB*, *PCDH7*, *PPIL1*, and *SRPK1* have already been reported to play an important role in cancer pathogenesis. For example, silencing *ABCE1* by small interfering RNA can inhibit the proliferation and invasiveness of small cell lung cancer cell lines [34]. *CKS1B*-depleted breast cancer cells not only exhibit slowed G(1) progression, but those cells also accumulate in G(2)-M due to blocked mitotic entry [35]. *HNRNPAB* was found to be overexpressed in highly metastatic cells and tumor tissues from patients with hepatocellular carcinoma with recurrence [36]. *PCDH7* was up-regulated in bone metastatic breast cancer tissues, and suppression of *PCDH7* inhibited breast cancer cell proliferation, migration, and invasion in vitro [37]. *PPIL1* was observed to be frequently overexpressed in colon cancer cells compared with noncancerous epithelial cells of the colon mucosa [38]. More interestingly, aberrant *SRPK1* expression in either direction might promote cancer by interfering with PHLPP-mediated dephosphorylation of Akt [39]. In this study, we demonstrated that even though the prognostic power of the 17-gene signature is superior, the individual genes, such as *HNRNPAB*, *PPIL1*, and *SRPK1,* can be used as individual biomarkers to predict recurrence-free survival.

### Resampling test for the 17-gene signature

**Figure 3:**
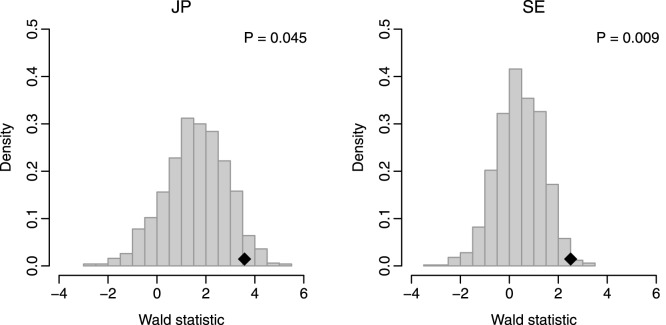
Resampling test for the 17-gene signature. The black rhombuses indicate the Wald statistics of the 17-gene signature. The grey histograms show the distribution of the Wald statistics for the 1,000 resampled gene signatures with identical size under the null hypothesis of no association between the 17-gene signature and recurrence-free survival.

We conducted a resampling test to determine whether the predictive power of the 17-gene signature was significantly better than that of random gene sets. We constructed 1,000 random gene signatures, each containing 17 genes that were randomly chosen from the human genome. The recurrence scores were calculated based on the randomized gene signatures, and univariate Cox proportional hazards regression of survival was conducted for each resampled gene signature. The association between each random gene signature and recurrence-free survival was measured using the Wald statistic. Our alternative hypothesis was that the Wald statistic value of our 17-gene signature should be higher than that of the randomized gene signatures if the 17-gene signature was more predictive than the randomized signatures. Fig. 3 indicates that the Wald statistic of the 17-gene signature was significantly higher than that of the randomized gene signatures (*P* = 0.045 for the JP cohort and *P* = 0.009 for the SE cohort), which suggests that the association between the 17-gene signature and recurrence-free survival is not by chance.

A previously published study by Venet et al. compared 47 prognostic breast cancer signatures to signatures of random genes [40]. It was found that ∼60% of the signatures were not significantly better outcome predictors than randomized signatures of identical size, and ∼23% were worst predictors than the median random signature [40]. Therefore, it is not possible to conclude that a particular mechanism is associated with human cancer from the finding that a gene signature for this mechanism predicts cancer outcome because most randomized signatures do [40]; however, here, we indicate that our 17-gene signature overcomes this problem.

### Multivariate analysis

We investigated the performance of the 17-gene signature in comparison with clinical factors associated with lung cancer outcome. For the JP cohort, we considered factors including patient age, gender, smoking history, stage, *EGFR*/*KRAS*/*ALK* gene alteration status, and Myc protein level. In the JP cohort, stage and *EGFR*/*KRAS*/*ALK* gene alteration status can individually predict recurrence-free survival (S2 Fig.). For the SE cohort, we took age, gender, and stage into account. However, none of these factor in the SE cohort can individually predict recurrence-free survival. A multivariate Cox proportional hazards regression of survival indicated that the 17-gene signature status remained a significant covariate in relation to the clinical factors in each validation cohort (*P* = 3.4×10^−3^ for the JP cohort and *P* = 2.5×10^−2^ for the SE cohort) (Table 3). In the JP cohort, patient age, stage, and *EGFR*/*KRAS*/*ALK* alteration status were also significant variables. However, in the SE cohort, the 17-gene signature status was the only significant covariant in the multivariate model (Table 3). These results strongly suggest that the 17-gene signature is largely independent of the traditional clinical factors and enhances the identification of lung cancer patients at greater risk for recurrence.

**Table 3:** Multivariate Cox proportional hazards regression of survival in the validation cohorts.

Cohort	Factors	Hazard ratio	95% Confidence interval	*P*-value
JP	17-gene + vs. −	2.35	(1.33, 4.16)	3.4×10^−3^
	Age (per year)	1.04	(1.01, 1.08)	2.4×10^−2^
	Gender male vs. female	0.79	(0.41, 1.54)	4.9×10^−1^
	Smoking + vs. −	1.34	(0.70, 2.58)	3.7×10^−1^
	Stage	2.59	(1.52, 4.41)	4.4×10^−4^
	Gene alteration + vs. −	0.51	(0.30, 0.86)	1.2×10^−2^
	Myc level high vs. low	0.81	(0.31, 2.11)	6.7×10^−1^
SE	17-gene + vs. −	2.03	(1.09, 3.76)	2.5×10^−2^
	Age (per year)	1.00	(0.96, 1.03)	8.3×10^−1^
	Gender male vs. female	0.91	(0.50, 1.64)	7.5×10^−1^
	Stage	1.50	(0.70, 3.23)	3.0×10^−1^

The 17-gene signature was derived from a “hypothesis-driven” method instead of whole genome screening. Traditionally, the prognostic power of the individual genes within human genome was tested one by one. The genes with the best statistical significance would be retained and used as cancer biomarkers. However, statistically-derived gene signatures by whole genome screening are often highly accurate in the discovery cohorts from which they were identified, yet most of them have not been validated as useful clinical tools [41], [42]. In this study, we first hypothesized that TNF-α is implicated in lung cancer. Then we pre-identified the genes that are potentially mediated by TNF-α/TNFR using TNF-α/TNFR KO mice. Multivariate analysis indicates that this “bottom-up” method yields a gene set with promising predictive power, which adds prognostic value to clinical and pathological findings in lung cancer.

## Conclusions

We investigated the gene expression profiles of two independent TNF-α/TNFR KO murine models. The EGFR signaling pathway was found to be the top pathway associated with genes mediated by TNF-α. Based on the TNF-α-mediated genes found in the murine models, we developed a prognostic gene signature that effectively predicted recurrence-free survival in lung cancer in two validation cohorts. When working cooperatively with known traditional clinical factors, the 17-gene signature may enhance prediction accuracy for identifying patients at higher risk for recurrence.

## Methods

### Microarray data processing

All the microarray data analyzed in this study were obtained from the GEO database [18]. The GC robust multichip average (GCRMA) algorithm [43] was used to summarize the expression level of each probe set for the microarray data. The significance analysis of microarrays (SAM) algorithm [44] was used to identify the differentially expressed genes between WT and TNF-α/TNFR KO mice. A paired t-test was used to detect the differentially expressed genes between the normal and tumor human lung tissues. *P*-values were adjusted using the Benjamini-Hochberg procedure.

### Risk scoring system

For the training cohort (KR), univariate Cox proportional hazards regression was used to evaluate the association between recurrence-free survival and gene expression. A recurrence score was then calculated for each patient using a linear combination of gene expression that was weighted by the Wald statistic (ratio of the regression coefficient to its standard error), as shown below [16], [29], [30], [31]:
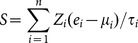



Here, *S* is the recurrence score of the patient; *n* is the number of genes; *Z_i_* denotes the Wald statistic of gene *i* (listed in Table 1); *e_i_* denotes the expression level of gene *i*; and *µ_i_* and *τ_i_* are the mean and standard deviation of the gene expression values for gene *i* across all samples. Patients were then divided into positive and negative groups, with the median of the recurrence score as the cutoff. A higher recurrence score implied a poor outcome. The scoring system and the associated scaling coefficients were fixed based on the training cohorts and then evaluated in the validation cohorts [16], [29], [30], [31].

### Statistical tool

All the statistical analyses were conducted by the R platform. The “survival” library was used to do survival analysis. The statistical significance between two Kaplan-Meier curves was determined by log-rank test using the “survdiff” function. Both univariate and multivariate Cox proportional hazards regression was conducted by the “coxph” function.

## Supporting Information

S1 Fig **The expression of** ***HNRNPAB*** **,** ***PPIL1*** **, and** ***SRPK1*** **predicts recurrence-free survival individually.** The patients in the JP cohort were stratified into two groups according to the expression level of each gene, using the median as a cutoff. The black curves are for the patients the gene expression higher than the median, while the gray curves are for the other patients.
(PDF)

S2 Fig **Stage and** ***EGFR*** **/** ***KRAS*** **/** ***ALK*** **gene alteration status individually predict recurrence-free survival in the JP cohort.** The left panel is for stage. The black curve is for the patients with stage II, while the gray curve is for the patients with stage I. The right panel is for gene alteration status. The black curve is for the patients without alteration, while the gray curve is for the patients with alteration. *P*-values were calculated using log-rank tests for the differences in survival.
(PDF)

S1 Table **The genes commonly differentially expressed between WT and TNF-α/TNFR KO mice.**
(XLSX)

S2 Table **The top 10 PANTHER pathways associated with the dysregulated genes in TNF-α/TNFR KO mice.**
(XLSX)

S3 Table **The TNF-α-mediated genes that are differentially expressed between the normal and tumor tissues.**
(XLSX)

S4 Table **The GO biological process terms associated with the TNF-α-mediated genes that are dysregulated in tumor tissues.**
(XLSX)
