# Supplementary figures and images for: Expression of Tumor Necrosis Factor-Alpha-Mediated Genes Predicts Recurrence-Free Survival in Lung Cancer
Source: PLoS One. 2014 Dec 30;9(12):e115945. doi: 10.1371/journal.pone.0115945 (PMC4280165; doi:10.1371/journal.pone.0115945)

HNRNPAB

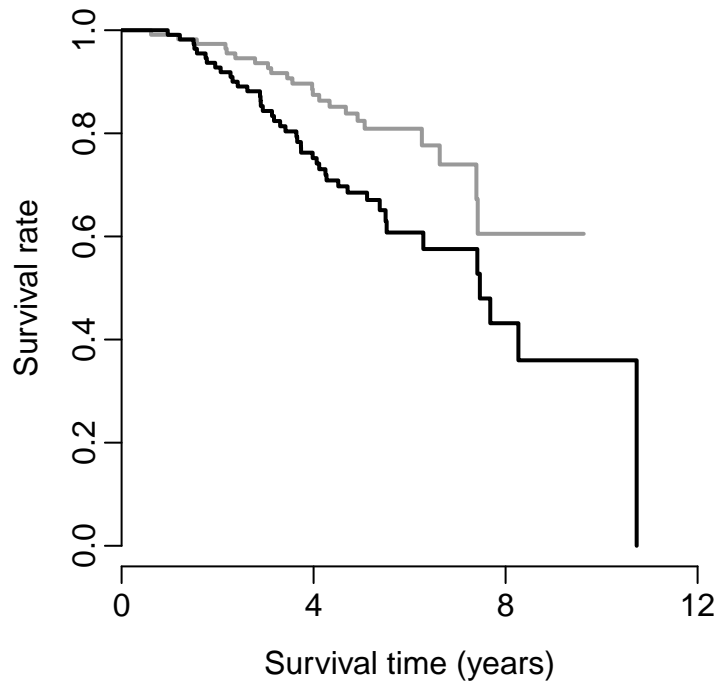

PPIL1

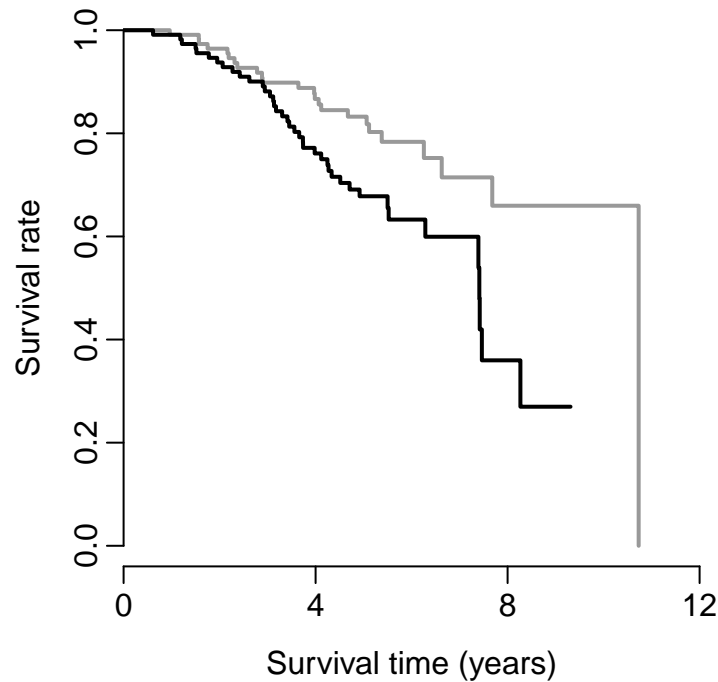

SRPK1

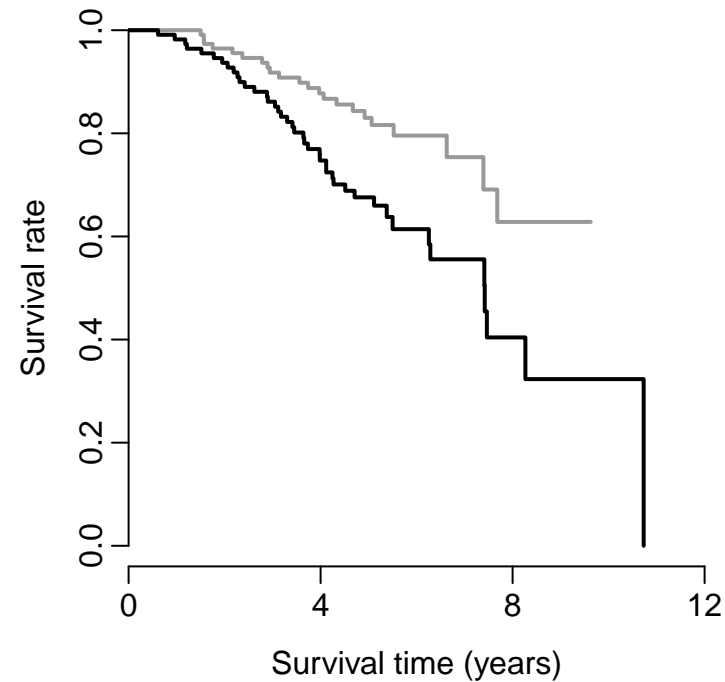

Supplement: S1 Fig — The expression of HNRNPAB , PPIL1 , and SRPK1 predicts recurrence-free survival individually. The patients in the JP cohort were stratified into two groups according to the expression level of each gene, using the median as a cutoff. The black curves are for the patients the gene expression higher than the median, while the gray curves are for the other patients. (PDF) [file pone.0115945.s001.pdf]

Stage

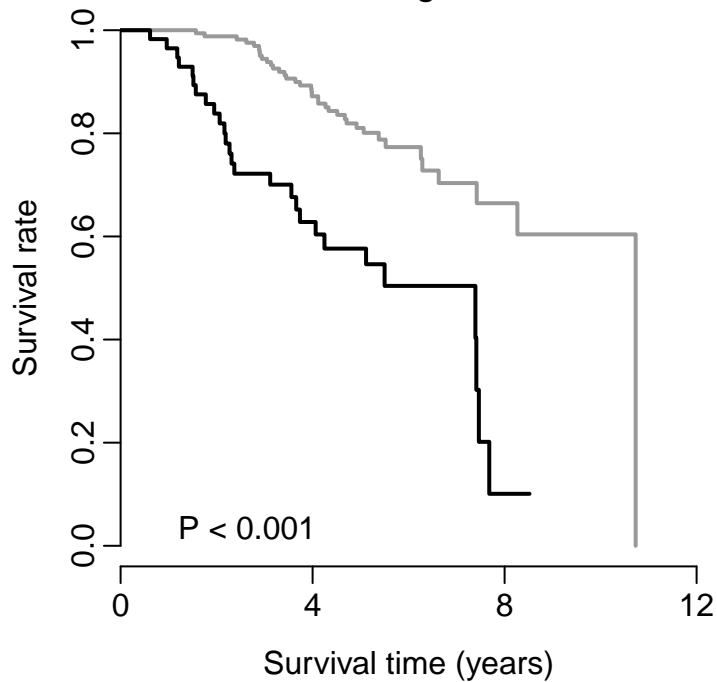

Gene alteration

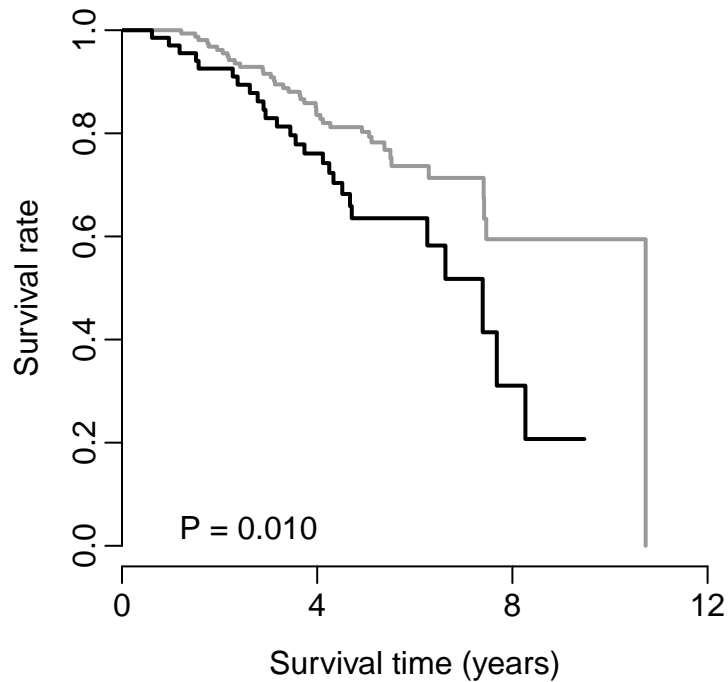

Supplement: S2 Fig — Stage and EGFR / KRAS / ALK gene alteration status individually predict recurrence-free survival in the JP cohort. The left panel is for stage. The black curve is for the patients with stage II, while the gray curve is for the patients with stage I. The right panel is for gene alteration status. The black curve is for the patients without alteration, while the gray curve is for the patients with alteration. P-values were calculated using log-rank tests for the differences in survival. (PDF) [file pone.0115945.s002.pdf]
